# Development of *Atropa belladonna* L. Plants with High-Yield Hyoscyamine and without Its Derivatives Using the CRISPR/Cas9 System

**DOI:** 10.3390/ijms22041731

**Published:** 2021-02-09

**Authors:** Lingjiang Zeng, Qiaozhuo Zhang, Chunxue Jiang, Yueyue Zheng, Youwei Zuo, Jianbo Qin, Zhihua Liao, Hongping Deng

**Affiliations:** 1 Chongqing Key Laboratory of Plant Resource Conservation and Germplasm Innovation, SWU-TAAHC Medicinal Plant Joint R&D Centre, School of Life Sciences, Southwest University, Chongqing 400715, China; zengling@swu.edu.cn (L.Z.); zhangqz2016@email.swu.edu.cn (Q.Z.); jiangchunxue@email.swu.edu.cn (C.J.); zhengyueyue@email.swu.edu.cn (Y.Z.); zuoyw1995@email.swu.edu.cn (Y.Z.); 2 Chongqing Academy of Science and Technology, Chongqing 401123, China; qjb1224@163.com

**Keywords:** *Atropa belladonna* L., CRISPR/Cas9, hyoscyamine 6*β*-hydroxylase, tropane alkaloids, transgenic plant

## Abstract

*Atropa belladonna* L. is one of the most important herbal plants that produces hyoscyamine or atropine, and it also produces anisodamine and scopolamine. However, the *in planta* hyoscyamine content is very low, and it is difficult and expensive to independently separate hyoscyamine from the tropane alkaloids in *A. belladonna*. Therefore, it is vital to develop *A. belladonna* plants with high yields of hyoscyamine, and without anisodamine and scopolamine. In this study, we generated *A. belladonna* plants without anisodamine and scopolamine, via the CRISPR/Cas9-based disruption of hyoscyamine 6*β*-hydroxylase (*AbH6H*), for the first time. Hyoscyamine production was significantly elevated, while neither anisodamine nor scopolamine were produced, in the *A*. *belladonna* plants with homozygous mutations in *AbH6H*. In summary, new varieties of *A. belladonna* with high yields of hyoscyamine and without anisodamine and scopolamine have great potential applicability in producing hyoscyamine at a low cost.

## 1. Introduction

*Atropa belladonna* L. is an important herbal plant used by human beings. Modern pharmaceutical science has revealed that *A*. *belladonna* plants produce anticholinergic tropane alkaloids (TAs), including hyoscyamine, anisodamine, and scopolamine [1]. Atropine is the racemic mixture of hyoscyamine, and its name derives from *A*. *belladonna*, which is widely cultivated to produce atropine or hyoscyamine [2,3,4]. Hyoscyamine, anisodamine, and scopolamine are clinically used for their anticholinergic properties. However, each of the three alkaloids is specifically used for the treatment of different disorders. Hyoscyamine is useful for the treatment of arrhythmias and organophosphate poisoning; anisodamine has been applied to treat infective shock, gastrointestinal colic and vascular spasm; scopolamine is well-known for curing motion sickness [5,6,7]. Medicinal plants are the only resource for the commercial production of these TAs. *A. belladonna* plants produce hyoscyamine as a major compound of TAs, and also produce the derivatives of hyoscyamine, including anisodamine and scopolamine, as minor compounds [6]. Therefore, it is necessary for the pharmaceutical industry to separate one compound from the others. For example, hyoscyamine has to be purified from raw extracts of *A*. *belladonna*, which contain hyoscyamine, anisodamine, and scopolamine in various mixtures. Due to their similar structures, it is difficult and expensive to independently extract hyoscyamine from the raw materials of *A*. *belladonna* that contain anisodamine and scopolamine. As such, it is important to develop *A*. *belladonna* plants without anisodamine and scopolamine, facilitating the separation of hyoscyamine at a low cost. Additionally, the low in planta content of hyoscyamine limits its supply and raises its price; therefore, the pharmaceutical industry is seeking to develop new varieties that produce hyoscyamine at high levels.

Hyoscyamine 6*β*-hydroxylase (H6H, EC 1.14.20.13) is a bifunctional dioxygenase, which catalyzes the 6*β*-hydroxylation of hyoscyamine to produce anisodamine, and subsequently converts anisodamine to scopolamine through epoxidation [8]. The *H6H* gene has been successfully employed to generate novel germplasm of *A*. *belladonna* with a high yield of scopolamine through metabolic engineering methods. The overexpression of the *H6H* gene from *Hyoscyamus niger* (*HnH6H*) markedly increased the production of scopolamine in *A*. *belladonna* plants [6] and in the hair root cultures of other TA-producing plants, such as *Hyoscyamus niger*, *Atropa baetica*, and *Scopolia lurida* [9,10]. Importantly, hyoscyamine was almost completely converted to scopolamine in the leaves of some *HnH6H*-overexpressing *A*. *belladonna* lines [6,11]. Inducing the overexpression of *H6H* is an efficient method to develop new varieties of *A*. *belladonna* that produce scopolamine at higher levels, and this method also markedly reduces hyoscyamine content. The *H6H* gene of *A*. *belladonna* has also been identified, and it can be used as the target gene for the generation of novel germplasm of *A*. *belladonna* without anisodamine and scopolamine via genome editing technologies.

Genome editing technologies are powerful tools for studying gene function and developing novel germplasm of plants. Categorized according to the genome-editing nuclease types, there are four groups of genome-editing technologies, including the CRISPR/Cas system, transcription activator-like effector nucleases (TALENs), zinc-finger nucleases (ZFNs), and meganucleases (MNs). All of the genome editing technologies can generate double-strand breaks (DSBs) in a specific DNA sequence of the genome [12,13,14,15,16]. DSBs can be efficiently repaired in two ways—non-homologous end joining (NHEJ) and homology-directed repair (HDR). NHEJ usually results in nucleotide insertion or deletion (indels), leading to a loss of or change in gene function. Of these genome-editing technologies, the CRISPR/Cas9 system is the most popular because of its advantages, such as its ease of use, its high efficiency, and its capacity for adaptation to diverse organisms [17]. The CRISPR/Cas9 system has been used to edit genes in various plants in order to generate new varieties [18,19,20], but it has not been tested in *A*. *belladonna*. In order to establish germplasm of *A*. *belladonna* without anisodamine and scopolamine, we used the CRISPR/Cas9 system to genetically edit the *H6H* gene, and then analyzed the production of tropane alkaloids in planta.

## 2. Results and Discussion

### 2.1. Cloning and Analysis of the Genomic Sequence of A. belladonna H6H Gene

**Figure 1:**
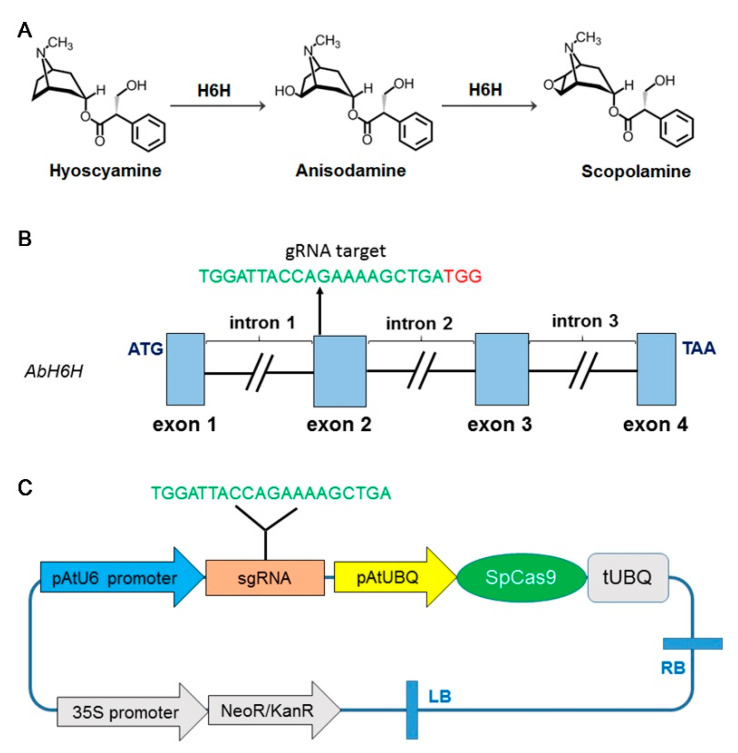
Catalytic function of hyoscyamine 6*β*-hydroxylase, genomic structure of *AbH6H*, and construction of the CRISPR/Cas9 expression vector, named pCas9-H6H. (**A**) Hyoscyamine 6*β*-hydroxylase catalyzes the 6*β*-hydroxylation of hyoscyamine to produce anisodamine, and subsequently converts anisodamine into scopolamine through epoxidation. (**B**) Genomic structure of *AbH6H* and the CRISPR/Cas9 target site in exon 2. The target region is shown in green letters followed by a red protospacer-adjacent motif (PAM), and black breaks between the exons indicate introns; (**C**) CRISPR/Cas9 expression vector construction. The sgRNA was driven by the *Arabidopsis thaliana* U6 promoter (pAtU6) with kanamycin resistance.

Hyoscyamine 6*β*-hydroxylase (H6H, EC 1.14.20.13) catalyzes the 6*β*-hydroxylation of hyoscyamine to form anisodamine, which is subsequently converted into scopolamine through epoxidation [8] (Figure 1A). The cDNA of *A*. *belladonna* H6H gene (*AbH6H*, GenBank number: JN415637) was reported in GenBank [21], and this helped us to identify its corresponding genomic DNA sequence from the genome of *A*. *belladonna*, sequenced by our group (data not released). To verify the consistency of the sequence, the coding sequence (CDS) and corresponding genomic DNA of *AbH6H* were obtained by PCR amplification and confirmed by a second process of sequencing. Subsequently, to analyze the structure of the *AbH6H* gene, we input the sequenced CDS and genomic DNA sequence into the NCBI (National Center for Biotechnology Information) website. The 1032-bp *AbH6H* CDS we obtained in this study was the same as the reported one (JN415637). The result showed that *AbH6H* was a split gene, containing four exons and three introns (Figure 1B). The total coding region was only 1032 bp, while the non-coding region was 2025 bp. In total, the genomic DNA sequence was 3057 bp, including the start codon and the stop codon. This conformed to the characteristics of eukaryotic genomes, which have more non-coding regions than coding regions. The analysis of genome structure was helpful for us in designing single guide RNA (sgRNA).

### 2.2. sgRNA Design and Vector Construction

The sgRNA that targeted *AbH6H* was designed with an online tool (http://crispor.tefor.net/) in December of 2018. The genome data of *Capsicum annuum* (*Solanaceae* plants) were used because *A. belladonna*’s genome sequence data were not available in this web tool. Potential sequences with high scores were screened. All potential 20 bp sequences followed by a protospacer-adjacent motif (PAM) in the open reading frame (ORF) of *AbH6H* were scored and analyzed based on several factors, including mismatches and the number of off-target sites. The employed guide ended with TTC or TTT, or contained only T and C in the last four nucleotides, and more than two Ts or at least one TT and one T or C (“TT-motif”). These guides should be avoided in polymerase III (Pol III)-based gene editing experiments that require high sgRNA expression levels [22]. The guide contained the sequence TTTT. It could not be transcribed with a U6 or U3 promoter, as TTTT terminates the transcription [22]. We chose as the guide sequence the sequence at the second exon, with a specificity score of 86, containing two restriction enzymes: *AluB*I and *Smo*I. This sequence could be transcribed with a U6 or U3 promoter, and ends without TTC or TTT (Figure 1C).

In the CRISPR/Cas9 vector, the *Streptococcus pyogenes* Cas9 (SpCas9) was driven by the cauliflower mosaic virus (CaMV) 35S promoter, and the sgRNA was driven by the U6-26 promoter of *A. thaliana*. To express this vector in medicinal plants, the selection marker gene *HPT* was replaced with *NPTII*. The 20 bp guide sequence from the *AbH6H* gene was inserted into the CRISPR/Cas9 vector to generate the Cas9-H6H construct. The Cas9-H6H vector was introduced into the *A. tumefaciens* strain EHA105 [23] using the freeze–thaw method [24].

### 2.3. Generation of Transgenic Atropa belladonna Plants

**Figure 2:**
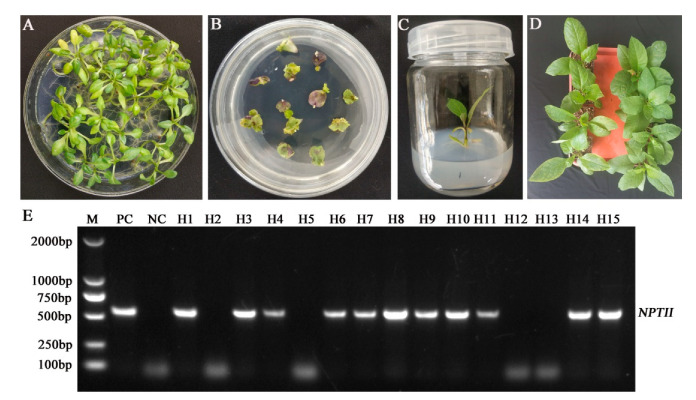
Generation and identification of transgenic *Atropa belladonna* plants. (**A**) Sterile *Atropa belladonna* plants. (**B**) Kanamycin-resistant plantlets induced from cotyledons. (**C**) Rooting of transgenic plants. (**D**) Transgenic plants in plastic pots. (**E**) PCR analysis of kanamycin-resistant *Atropa belladonna* transgenic plants. M, 2 kb molecular weight marker; PC, plasmid pCas9-H6H; NC, non-transgenic *Atropa belladonna*; H1–H15, transformed *Atropa belladonna* plants.

Sterile seedlings of *A*. *belladonna* were generated (Figure 2A) and their cotyledons were used as initial explants for genetic transformation. After the cotyledons of *A. belladonna* were infected with the engineered *A*. *tumefaciens* EHA105 harboring the plant-expressing vector pCas9-H6H, some cotyledons gradually turned white or brown and became necrotic after the subculture stage; some cotyledons in the selection medium with kanamycin generated new shoots from the wounded sites within four weeks of the genetic transformation (Figure 2B). When the regenerated seedlings grew to 2 cm in height, they were transferred to, and cultured on, a rooting medium (MS with 0.20 mg/L indole butyric acid and 200 mg/L cephalosporin) (Figure 2C). After 2–3 weeks, when the regenerated plants had formed roots, they were transferred into plastic pots and grown in a greenhouse under a 16 h light/8 h dark photoperiod cycle at 25 °C (Figure 2D).

PCR was used to detect the *NPTII* gene in *A. belladonna* plants. A 502-bp DNA fragment specific to the marker gene (*neomycin phosphotransferase II*, *NPTII*) was amplified from the pCas9-H6H plasmid (positive control) and transgenic plants of *A*. *belladonna*, and was not amplified from wild type *A. belladonna* plants (negative control) (Figure 2E). The PCR results were consistent with previous reports, in which *NPTII* was also used as the marker gene detected by the same primers [11]. The PCR detection results indicated that T-DNA harboring the *AbH6H* target DNA sequence was integrated into the genome of *A. belladonna*, and of the 15 generated plants, there were 11 transgenic plants, suggesting that some generated plants were able to resist kanamycin at the concentration used in this study.

### 2.4. Detection of CRISPR/Cas9-Mediated Mutagenesis in AbH6H

**Figure 3:**
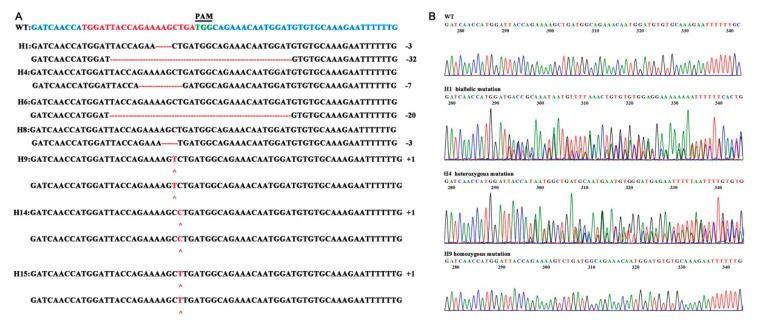
Sequences of the genomic DNA extracted from the transgenic mutant *Atropa belladonna* plant lines. (**A**) The wild type (WT) sequence appears at the top, with the PAM (TGG) shown in bold green text. DNA insertions and point mutations are added under each sequence in red. Deletions are shown as dashes, and insertions are shown as plus signs. The indel size shows the gain/loss in the amplicon length in the target region. (**B**) Representative chromatograms of WT, biallelic mutation (H1), heterozygous mutation (H4) and homozygous mutation (H9) lines.

To investigate CRISPR/Cas9-mediated mutagenesis in *AbH6H*, PCR amplifications were carried out using the primer pair AbH6H-knock-F/R flanking the designated target site (Table 1). The PCR products were sequenced directly using the primer AbH6H-knock-F, the favorable binding positions of which are 269 bp upstream of the target site. The sequencing results were directly decoded using the degenerate sequence decoding method [25]. Generally, the CRISPR/Cas9-mediated mutagenesis includes biallelic, heterozygous and homozygous mutation. Biallelic and heterozygous mutation produces the se overlapping peaks of chromatogram, while homozygous mutation does not [17,25]. Of the 11 transgenic plants, there were four plants exhibiting editing and seven plants exhibiting no editing. The mutation rate was approximately 63.6% in 11 transgenic plants for *Cas9-H6H* (Figure 3A). The sequencing results suggest that three transgenic plant lines (H9, H14, H15) among the transgenic lines (27.3%, 3/11) were homozygous, with mutations occurring in the same DNA locus in both alleles, both of which were inserted by one base. Others lines exhibited non-homozygous mutations, including one biallelic mutation line (H1; two distinct variations) and three heterozygous mutations (H4, H6, and H8; single mutation) (Figure 3B).

The results show that most of the transgenic plants tested had insertions or deletions, a few of which were off-target. The results were a little different from those of previous reports concerning *A. thaliana*, probably because of the selection of target DNA. Among the seven transgenic plants, all kinds of mutation-containing sequences caused changes in the majority of amino acids and frameshifts in the ORF. These homozygous lines, H9, H14, and H15, were also micro-propagated for alkaloid analysis.

### 2.5. Analysis of Tropane Alkaloids

**Figure 4:**
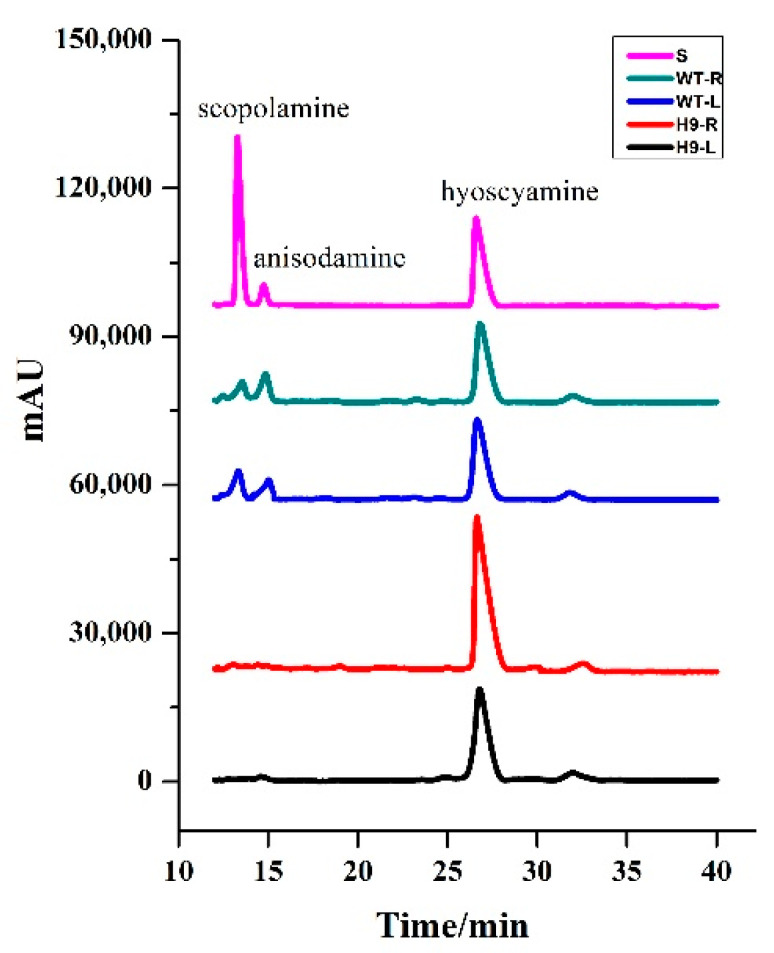
HPLC trace of tropane alkaloids in *Atropa belladonna* plants. S, standard samples; WT-R, roots of wild type plants; WT-L, leaves of wild type plants; H9-R, roots of transgenic plants; H9-L, leaves of transgenic plants.

To investigate the effects of the CRISPR/Cas9-mediated disruption of *AbH6H* on alkaloid production, we analyzed pharmaceutical TAs, including hyoscyamine, anisodamine, and scopolamine, in the roots and leaves of *A*. *belladonna*. The HPLC trace of alkaloids indicated that the retention times of authentic hyoscyamine, anisodamine, and scopolamine were 26.59 min, 14.75 min, and 13.28 min, respectively (Figure 4). Peaks respectively corresponding to hyoscyamine, anisodamine, and scopolamine were also detected in alkaloid extracts of the wild type *A*. *belladonna*’s leaves and roots. The hyoscyamine peak was also detected in alkaloid extracts from *AbH6H*-disrupting plants, while the peaks of anisodamine and scopolamine were not detected in *AbH6H*-disrupting plants, indicating that the conversion from hyoscyamine to anisodamine and scopolamine was completely disrupted. The alkaloid analysis results confirm that the CRISPR/Cas9-mediated disruption of *AbH6H* led to a loss of function in the H6H in transgenic lines with homozygous mutations, including H9, H14, and H15. Since the H6H-catalyzed reactions were disrupted completely, attention should be paid to the hyoscyamine production in planta.

**Figure 5:**
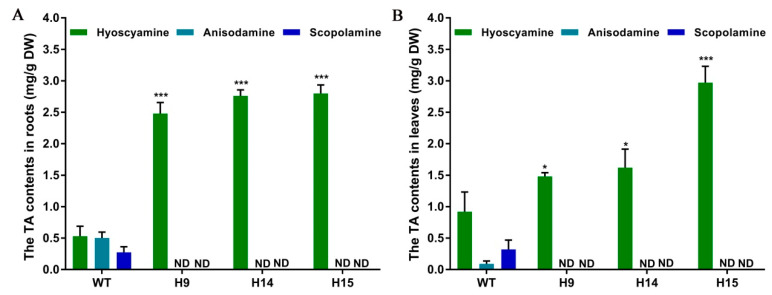
The contents of tropane alkaloids in (**A**) roots and (**B**) leaves of *Atropa belladonna*. WT, wild type *Atropa belladonna* plants; H9, H14, H15, independent transgenic plants with knockout of *AbH6H*. * and *** represent significant difference (given by t-test) at the levels of *p* < 0.05 and *p* < 0.001, respectively. ND, not detected.

Wild type *A*. *belladonna* plants produced hyoscyamine, anisodamine, and scopolamine at the levels of 0.53 mg/g dry weight (DW), 0.50 mg/g DW, and 0.27 mg/g DW in their roots, and at the levels of 0.92 mg/g DW, 0.09 mg/g DW, and 0.32 mg/g DW in their leaves. The hyoscyamine levels were much higher than the scopolamine levels in the roots and leaves of wild type plants, indicating that hyoscyamine is the main alkaloid in *A*. *belladonna* [11]. In the roots of the H9, H14, and H15 plants, the hyoscyamine contents were 2.48 mg/g DW, 2.76 mg/g DW, and 2.80 mg/g DW, respectively. The hyoscyamine content was increased 3.68-, 4.21-, and 4.28-fold in the roots of the three lines with homozygous mutations of *AbH6H* compared to in the roots of wild type plants (Figure 5A). In the leaves of H9, H14, and H15, the hyoscyamine contents were 1.48 mg/g DW, 1.62 mg/g DW, and 2.97 mg/g DW, respectively, and were increased 0.61-, 0.76-, and 2.22-fold, respectively, compared to the levels in the leaves of wild type plants (Figure 5B). Generally, substrates are able to accumulate at higher levels when the biosynthesis genes downstream from them are suppressed or disrupted. The suppression or silencing of TA biosynthesis genes, such as ornithine decarboxylase [26], tropinone synthase [27], phenylpyruvic acid reductase [28], and littorine synthase [29], dramatically reduced the corresponding alkaloid production, while markedly increasing the accumulation of related substrates and precursors in *A*. *belladonna*. Because anisodamine and scopolamine are the products of H6H catalysis, the CRISPR/Cas9-mediated loss of H6H function halted the conversion of hyoscyamine into its products, and, thus, the hyoscyamine accumulation level was increased.

In conclusion, we established *Atropa belladonna* plant lines with reduced hyoscyamine 6*β*-hydroxylase function by using CRISPR/Cas9 systems for the first time. The novel germplasm lines with homozygous mutations in *AbH6H* produced hyoscyamine at higher levels, and, in these lines, anisodamine and scopolamine were not synthesized. The novel germplasm of *A*. *belladonna* has great potential applicability in developing new varieties that can produce hyoscyamine at a low cost.

## 3. Material and Methods

### 3.1. Cloning and Analysis of the Genomic Sequence of the H6H Gene of A. belladonna

In order to verify the consistency between the coding sequence of *AbH6H* and its corresponding genomic DNA sequence, we isolated the coding sequence of *AbH6H* using total cDNA as the template, and the genomic DNA sequence of AbH6H using genomic DNA as the template. Total RNA was extracted from the secondary roots of *A. belladonna* using an RNAsimple Total RNA Kit (TIANGEN, Beijing, China), and then cDNAs were synthesized using a FastKing RT Kit (TIANGEN, Beijing, China). The reported cDNA sequence of *AbH6H* (GenBank Number: JN415637) was obtained from GenBank and then amplified using a pair of primers, FH6H and RH6H, using total cDNA as the template. Genomic DNA of *A. belladonna* was extracted using the CTAB method [30]. The two primers, FH6H and RH6H, were also used to amplify the genomic DNA of *AbH6H* using the genomic DNA of *A*. *belladonna* as the template. The amplification mixture contained 5 μL 10 × KOD Buffer, 5 μL dNTPs (2 mM), 2 μL MgCl2 (25 mM), 1 μL FH6H (10 μM), 1 μL RH6H (10 μM), 1 μL KOD plus Polymerase (1.0 U/μL), 1μL DNA (100 ng/μL) or cDNA template, and water added to achieve a total volume of 50 μL. The PCR program was as follows: 94 °C for 5 min, 1 cycle; 94 °C for 30 s, 56 °C for 30 s, 68 °C for 1 kb/min, 32 cycles; 68 °C for 5 min, 1 cycle. The amplified sequences were inserted into the pMD19-T vector (Takara, Beijing, China) and then sequenced by the company (Tsingke, Chengdu, China). A comparison analysis between the coding sequence and genomic DNA sequence of *AbH6H* was performed online (https://www.ncbi.nlm.nih.gov/sutils/splign/splign.cgi) in November of 2018.

### 3.2. Design of sgRNA and Construction of CRISPR/Cas9 Vector

The derived coding sequence of the *AbH6H* gene was input into the relevant website (http://crispor.tefor.net/) in December of 2018 to analyze potential gene editing sites. This website evaluates all the possible sequences followed by 20-NGG, and ranks them from high to low in order to evaluate which sgRNA would be the best choice for editing based on the off-target possibilities and the location of the gene. Sequences with high marks are ideal targets for editing. The ideal target was chosen, and a pair of complementary oligos were synthesized by Tsingke (Chengdu, China) and annealed to generate the dimer. The hygromycin-resistant gene in the original pCAMBIA1300-Cas9 [31] was deleted using *Xho*I. Then, *NPTII* was inserted into the linearized pCAMBIA1300-Cas9 to generate a new construct, pCAMBIA1300-Cas9N. The dimer was ligated into a linearized pCAMBIA1300-Cas9N plant expression vector via *Bsa*I enzyme digestion and recombination, forming a new vector, pCas9-H6H.

### 3.3. Establishment of Transgenic Plants of Atropa belladonna

The *Atropa belladonna* seeds were germinated at 25 °C under a 16 h light/8 h dark photoperiod cycle. After 2 weeks, they were used for transformation. The pCas9-H6H was introduced into the *A. tumefaciens* strain EHA105 using the freeze–thaw method [24]. Co-cultivation was carried out in the dark at 25 °C for 4 days. After co-cultivation, the explants were transferred to the selection medium (the MS solid medium with 1 mg L^−1^ zeatin, 0.5 mg L^−1^ indole-3-acetic acid, 400 mg L^−1^ kanamycin and 200 mg L^−1^ cephalosporin). The method for transforming *Atropa belladonna* plants was based on the previous report, with slight differences [32]. When the transformed plants generated roots, they were transplanted into substrates composed of PINDSTRUP-moss:vermiculite:perlite (v:v:v = 3:6:1) and grown in the greenhouse under a 16 h light/8 h dark photoperiod cycle at 25 °C.

### 3.4. Molecular Analysis of Transformants

When the *A. belladonna* plants grew to 25 cm in height [26], the genomic DNA was isolated from the leaves of the transformed and untransformed *A. belladonna* plants using the TPS protocol [33]. PCR detection was conducted using a pair of primers (*NPTII*-F/R). Wild type *A. belladonna* plants were used as the negative control, and the pCas9-H6H vector was used as the positive control. The samples with positive PCR results were kept for further PCR detection with another pair of detection primers (AbH6H-Knock-F/R) and further sequencing. The amplification mixture contained 10 μL 2 × mixture buffer, 0.5 μL NPTII-F (10 μM), 0.5 μL NPTII-R (10 μM), 100 ng template DNA, and water added to achieve a total volume of 20 μL. The PCR program was as follows: 94 °C for 5 min, 1 cycle; 94 °C for 30 s, 56 °C for 30 s, 72 °C for 45 s, 35 cycles; 72 °C for 5 min, 1 cycle. The primers used in this study were listed in Table 1. The edited base was analyzed according to the sequenced results.

### 3.5. Alkaloid Detection

TAs were extracted from approximately 200 mg of dried plant material, according to a previously reported method [34]. The mobile phase was composed of acetonitrile and ammonium acetate (acetonitrile:50 mM ammonium acetate = 89:11). The flow speed was 1 mL/min. The standard samples of hyoscyamine, anisodamine, and scopolamine were purchased from Sigma-Aldrich (Sigma, LA, USA). The standard samples were dissolved in methanol at a final concentration of 2000 mg/mL and were gradually serially diluted to 1000, 750, 500, 250, 100, 50, 25, 10, and 5 mg/mL. The HPLC system was an LC-20A from Shimadzu; the detector was a photo-diode array. The detecting wavelength was 226 nm. The temperature of the CTP-ODS column (150 × 4.6 mm) was 40 °C. There were at least three independent biological replicates. The sample solution was injected at 20 μL each time. The method for the analysis of the TAs content was the same as that described in our recent work [26,28,29,35,36].

## Figures and Tables

**Table 1:** Primer information.

Primers	Purpose	Primer Sequence (5′-3′)
FH6H	Gene cloning	ATGGCTACTCTTGTCTCAAATTG
RH6H		TTAGGCATTAATTTTATATGGCTTAAC
NPTII-F (*Xho*I)	Vector construction	CGCCTCGAGATGATTGAACAAGATGGATTG
NPTII-R (*Xho*I)		CGCCTCGAGTCAGAAGAACTCGTCAAGAAG
AbH6H-F	Vector construction	GATTGTGGATTACCAGAAAAGCTGA
AbH6H-R		AAACTCAGCTTTTCTGGTAATCCAC
AbH6H-knock-F	PCR detection	CATGCGCATGATATGTGAAC
AbH6H-knock-R		CCGGATGAAGGCGATTCAG

## Data Availability

Not applicable.

## Abbreviations

PAM	protospacer-adjacent motif
NPTII	neomycin phosphotransferase II
